# Combined treatment of sodium ferulate, *n*‐butylidenephthalide, and ADSCs rehabilitates neurovascular unit in rats after photothrombotic stroke

**DOI:** 10.1111/jcmm.13894

**Published:** 2018-11-12

**Authors:** Yong‐Hua Zhao, Nai‐Wei Liu, Chien‐Chih Ke, Bo‐Wen Liu, Yi‐An Chen, Cheng Luo, Qian Zhang, Zhen‐Yan Xia, Ren‐Shyan Liu

**Affiliations:** ^1^ State Key Laboratory of Quality Research in Chinese Medicine Faculty of Chinese Medicine Macau University of Science and Technology Taipa Macau; ^2^ Biomedical Imaging Research Center National Yang‐Ming University Taipei Taiwan; ^3^ Department of Biomedical Imaging and Radiological Sciences National Yang‐Ming University Taipei Taiwan; ^4^ Molecular and Genetic Imaging Core/Taiwan Mouse Clinic National Comprehensive Mouse Phenotyping and Drug Testing Center Taipei Taiwan; ^5^ Department of Medical Imaging and Radiological Sciences Kaohsiung Medical University Kaohsiung Taiwan; ^6^ Institute of Clinical Medicine National Yang‐Ming University Taipei Taiwan; ^7^ Department of Biotherapy Shenzhen Luohu People's Hospital Shenzhen China; ^8^ Department of Nuclear Medicine Cheng‐Hsin General Hospital Taipei Taiwan; ^9^ Department of Nuclear Medicine and National PET/Cyclotron Center Taipei Veterans General Hospital Taipei Taiwan

**Keywords:** adipose‐derived stromal cells, *n*‐butylidenephthalide, neurovascular unit, rehabilitation, sodium ferulate

## Abstract

The remodelling of structural and functional neurovascular unit (NVU) becomes a central therapeutic strategy after cerebral ischaemic stroke. In the present study, we investigated the effect of combined therapy of sodium ferulate (SF), *n*‐butylidenephthalide (BP) and adipose‐derived stromal cells (ADSCs) to ameliorate the injured NVU in the photochemically induced thrombotic stroke in rats. After solely or combined treatment, the neovascularization, activation of astrocytes, neurogenesis, expressions of vascular endothelial growth factor (VEGF) and claudin‐5 were assessed by immunohistochemical or immunofluorescence staining. In order to uncover the underlying mechanism of therapeutic effect, signalling of protein kinase B/mammalian target of rapamycin (AKT/mTOR), extracellular signal‐regulated kinase 1/2 (ERK1/2), and Notch1 in infarct zone were analysed by western blot. ^18^F‐2‐deoxy‐glucose/positron emission tomography, magnetic resonance imaging, Evans blue staining were employed to evaluate the glucose metabolism, cerebral blood flow (CBF), and brain‐blood barrier (BBB) permeability, respectively. The results showed that combined treatment increased the neovascularization, neurogenesis, and VEGF secretion, modulated the astrocyte activation, enhanced the regional CBF, and glucose metabolism, as well as reduced BBB permeability and promoted claudin‐5 expression, indicating the restoration of structure and function of NVU. The activation of ERK1/2 and Notch1 pathways and inhibition of AKT/mTOR pathway might be involved in the therapeutic mechanism. In summary, we have demonstrated that combined ADSCs with SF and BP, targeting the NVU remodelling, is a potential treatment for ischaemic stroke. These results may provide valuable information for developing future combined cellular and pharmacological therapeutic strategy for ischaemic stroke.

## 1 INTRODUCTION

Although neuroprotective agents are demonstrated to be effective in functional recovery of stroke in animal model, most clinical trials showed uniformly negative results.^1^ It suggests that targeting only neuronal cells is not enough for stroke therapy and neuron‐astrocyte‐capillary interaction should be taken into consideration.^2^ In 2002, Report of the Stroke Progress Review Group updated the concept of neurovascular unit (NVU) which emphasized the complexity of interactions between neurons, vascular cells and glia in the brain. Since then, protection and regeneration of NVU has been a new focus for stroke treatment.^3^ Studies of NVU describe that upon brain injury, astrocytes regulate the neurovascular coupling by promoting angiogenesis and improve the regional cerebral blood flow (rCBF). In addition, the newly formed vessels secrete neurovascular trophic factors and chemokines in the microenvironment of injured brain that induces the migration of adjacent neural stem cells and subsequent integration in the parenchyma of ischaemic region. Coordination of neovasculature and astrocytes also regulate the synaptogenesis and axonal sprouting.^4^, ^5^ Therefore, NVU is now the important therapeutic target for postischaemic stroke treatment and management.

In last two decades, numerous reports have shown that mesenchymal stem cells (MSCs) are a promising therapeutic regimen of human diseases including cerebral ischaemia. Administration of adipose‐derived stromal cells (ADSCs), a type of MSCs, decreased the infarct volume, inflammation, cell apoptosis, and improved the poststroke sensorimotor dysfunction. Furthermore, stereotactically injected ADSCs were found to differentiate into neurons which replaced necrotic cells, and increased neuronal survival.^6^, ^7^ In a study of comparing the therapeutic effect of MSCs from adipose tissue and bone marrow (BM) on ischaemic stroke, ADSCs had distinctly higher proliferation, differentiation and secretory ability, and their efficacies of reducing the infarct size and brain oedema, improving the motor function were also superior to BM‐derived stem cells.^8^ In recent years, ADSCs have received great attention because of their nonethical controversy compared to embryonic stem cells, and minimally invasive collection procedure compared to BM‐MSCs. These advantages have made ADSCs a more appropriate and important source of MSCs in the development of cell therapy for human diseases including stroke.

Combined pharmaceutical treatment with MSCs for ischaemic stroke is a potential and feasible approach. More effective and synergistic therapeutic outcome for ischaemic stroke by combined MSCs and pharmaceutical treatment through promotion of stem cell migration and survival, anti‐apoptosis, endogenous stem cell proliferation, neurotrophic factor secretion, and angiogenesis have been observed.^9^ Among the drugs used in the combined treatment with stem cells, several Chinese medicines and their active compounds showed remarkable therapeutic results, such as to induce differentiation of MSCs into neural‐like cells, maintain the pluripotency of embryonic stem cells and enhance the efficacy of induced pluripotent stem cell generation.^10^, ^11^, ^12^ Our previous study also showed that sodium ferulate (SF) and *n*‐butylidenephthalide (BP), two active components extracted from Radix Angelica sinensis, were able to enhance angiogenesis, neurogenesis and reduce infarction volume in the rat middle cerebral artery occlusion (MCAo) model when used in combination with MSCs.^13^ However, the therapeutic effect of combined treatment with ADSCs, SF, and BP on the photothrombotic stroke (PTS) model is still unknown. In this study, with the aid of multimodal imaging techniques (eg, magnetic resonance imaging, ^18^F‐2‐deoxyglucose positron emission tomography and fluorescent optical imaging), we sought to know the efficacy of the combined therapy on restoring the structure and function of NVU in infarct region after stroke.

## 2 MATERIALS AND METHODS

All animal protocols have been carried out in accordance with ARRIVE (Animal Research: Reporting of In Vivo Experiments) guidelines and approved by the Institutional Animal Care and Use Committee of Macau University of Science and Technology and National Yang‐Ming University.

### 2.1 ADSCs isolation and identification

ADSCs were isolated from adipose tissue of six‐week‐old C57BL/6L mice. Briefly, adipose tissue was carefully excised from abdominal cavity and immediately digested by 0.1% collagenase type IV mixed in alpha‐minimum essential medium (αMEM) containing 10% foetal bovine serum (FBS, Hyclone) at 37°C for 1 hour. The digested tissue was filtered using a 45 μm nylon filter mesh (BD Falcon) and centrifuged at 1500 *g* for 10 minutes. After removal of the supernatant, the pellet was resuspended in αMEM supplemented with 20% FBS and seeded in a 6‐cm tissue culture dish. The culture medium was refreshed every 2 days, and cells were passaged at 80%‐90% confluence.

To identify the isolation of a true ADSC population, we evaluate the adipogenic and osteogenic differentiation potential of ADSCs. Cells at 80% confluence were incubated with adipogenic and osteogenic induction medium, respectively. Adipogenic induction medium contained 1 m mol L^−1^ dexamethasone, 0.5 m mol L^−1^ 3‐isobutyl‐1‐methyl‐xanthine, 10 μg/mL recombinant human insulin, 100 m mol L^−1^ indomethacin and 10% FBS. Osteogenic induction medium contained 100 n mol L^−1^ dexamethasone, 10 m mol L^−1^ β‐glycerophosphate, 0.2 m mol L^−1^ ascorbate, and 10% FBS. After 2 weeks of induction, cells were fixed with 4% formaldehyde (Sigma‐Aldrich) followed by staining with 0.18% Oil Red O (Sigma‐Aldrich) for 5 minutes or with 1% Alizarin‐red S (Sigma Aldrich) solution in water for 10 minutes. The control cells were cultured with maintenance medium and underwent the same staining procedures above.

### 2.2 Introduction of luciferase gene in ADSCs

Codon‐optimized reporter gene encoding firefly luciferase (Luc) was cloned in a lentiviral expressing vector. Lentiviral particles were produced by cotransfection with plasmid pRSV‐Rev, pMDLg‐PRRE, and pHEF‐VSVG into 293T packaging cells. 24 hours later, the medium was replaced by fresh medium containing 1% bovine serum albumin (BSA). Virus‐containing medium was collected 24 hours later and filtered with 22 μm filter. ADSCs were infected with lentiviral particles followed by puromycin selection (2 mg/mL) to establish stably transduced cells. The Luc expression in ADSCs was evaluated by incubating the colonies with luciferin solution and imaged with IVIS 50 (Perkin Elma, UK). Image parameter was set as following: Exposure Time = Auto, Binning = medium, f/stop = 1, FOV = 12. Quantification was performed using living image software 3.2 (IVIS Imaging System, Perkin Elma, UK).

### 2.3 Photochemically induced stroke model

The details of experimental method were described in our previous study.^14^ Briefly, 7‐week old male Sprague‐Dawley (SD) rats under anesthetization was illuminated by a laser beam with 532 nm wavelength at the middle of the craniotomic window which was made over the somatosensory cortex with the center at the coordinate of 1 mm rostrally from the bregma and 3.5 mm lateral to the midline for 30 minutes upon slow injection of rose bengal (20 mg/kg body weight) through tail vein. All rats after induced stroke were able to survive until they were killed at the end‐point in this study. Before and after thrombosis was induced by photochemical method, a moorFLPI‐2 Full‐Field Laser Perfusion Imager (Moor Instruments, Axminster, UK) was placed at the center of cranial window where the laser beam illuminated in order to examine the blockage of rCBF owing to cerebral vascular embolization. The laser speckle images were acquired with 25‐Hz sampling frequency, 1 frame/s, 580 × 752 pixels resolution, and zoom size of 5.6 mm × 7.5 mm.

### 2.4 Experimental groups and therapeutic interventions

Rats with induced photochemical stroke were randomly divided into four groups: PTS, SF + BP, ADSC, and ADSC + SF + BP. Four hours after stroke induction, 20 μL phosphate buffer saline (PBS) was injected into the margin of laser illuminated area in rats of group PTS and SF + BP. In the rats of group ADSC and ADSC + SF + BP, 5 × 10^5^ ADSCs (in 20 μL PBS) were injected into the same place. After stem cells or PBS injection, SF (60 mg/kg) was daily administrated in rats of group SF + BP and ADSC + SF + BP for consecutive 14 days via intraperitoneal injection. BP (10 mg/kg) was subcutaneously injected in the same groups once a day for 3 days. For control treatment in rats of group PTS and ADSC, PBS was injected following the time‐points of SF and BP delivery.

### 2.5 Immunohistochemical staining

After animals (n = 6) were killed, intact brains were removed from rats at day 7 and 14 after stroke induction. Brain tissue was then fixed, dehydrated, xylene clearing, paraffin‐embedded, and cut into 5‐μm section slices for subsequent staining. After one hour blocking (10% serum, 1% BSA, and 0.025% TritonX‐100 in tris‐HCL buffered solution [TBS]), sections were incubated overnight with primary antibodies (prediluted in TBS containing 1% BSA) at 4°C. After rinsed with TBS‐0.1% Tween‐20, tissue sections were detected using horseradish peroxidase‐conjugated secondary antibodies and the DAKO Dual Link system (DAKO, K4065) with 2% 3,3‐diaminobenzidine. The stained tissue was scanned using the Aperio CS2 (Leica) and three individual field of views at infarcted region in each sample slice were evaluated. Primary antibodies used in this study were listed as following: anti‐Luc (1:100, G745A, Promega), anti‐von Willebrand Factor (vWF, 1:100, ab6994, Abcam), anti‐glial fibrillary acidic protein (GFAP, 1:200, ab53554, Abcam), anti‐vascular endothelial growth factor (VEGF, 1:100, ab1316, Abcam), anti‐alpha smooth muscle actin (αSMA, 1:100, ab7817, Abcam) and anti‐neuron‐specific class III beta‐tubulin (Tuj‐1, 1:200, ab18207, Abcam).

### 2.6 Immunofluorescence staining

Brains of each group (n = 3) at day 3 and day 7 were fixed with fresh 4% paraformaldehyde solution, and cut into coronal sections of 10 μm thickness for immunofluorescence staining of claudin‐5 (1:200, 34‐1600, Invitrogen) at 4°C for overnight. Subsequently, brain samples were washed with PBS and incubated with the Alexa Fluor^®^ 488 goat anti‐rabbit immunoglobulin (Life Technologies) secondary antibodies for 1 hour at 37°C. Every group was digitally photographed with a confocal laser scanning microscope (Leica Microsystems Inc., Buffalo Grove, United States).

### 2.7 Western blot

Protein samples (n = 3) were, respectively, extracted from the infarct tissue at day 7 and ipsilateral hemispheres at day 14 after stroke induction. Western blot was performed according to previous protocol.^13^ Briefly, 20 μg total protein in sample buffer (4 ×  SDS loading buffer: 10% glycerol, 62.5 m mol L^−1^ Tris‐HCl pH 6.8, 1% SDS, 65 m mol L^−1^ DTT and bromophenol blue) was incubated in the boiling water for 10 minutes. Polyacrylamide gel electrophoresis (PAGE) was performed followed by electrotransfer of sample onto polyvinylidene difluoride (PVDF) membranes. After blocking with 5% (w/v) nonfat milk in PBS for 2 hours at room temperature, membranes were incubated with primary antibodies at 4°C overnight. Antibodies used in this study include doublecortin (DCX, 1:500, ab77450, Abcam) for the detection of neurogenesis after 14 days, and anti‐AKT (1:500, 4691s, Cell Signaling Technology), anti‐p‐AKT (1:500, 4060s, Cell Signaling Technology), anti‐mammalian target of rapamycin (mTOR, 1:500, 2983s, Cell Signaling Technology), anti‐p‐mTOR (1:500, 2971s, Cell Signaling Technology), anti‐ERK1/2 (1:500, 4695s, Cell Signaling Technology), anti‐p‐ERK1/2 (1:500, 4370s, Cell Signaling Technology), anti‐Notch1 (1:1000, 3608s, Cell Signaling Technology) for the detection of signal pathways after 7 days, as well as β‐actin (1:1000, A4700, Sigma). The membranes were gently washed with TBST (50 m mol L^−1^ Tris‐HCl [pH 7.4; Acros Organics BVBA, Geel, Belgium], 150 m mol L^−1^ NaCl, 0.05% Tween 20 [Acros Organics BVBA]) three times and incubated with goat anti‐rabbit IgG (H + L) secondary antibodies (1:5000, LI‐COR Biosciences) at room temperature for 1 hour. Chemiluminescent signal was imaged and quantified using the Odyssey Infrared Imager (LI‐COR).

### 2.8 Perfusion‐weighted imaging by MRI

Perfusion‐weighted imaging (PWI) was performed with the procedure described in our previous report.^14^ BioSpec‐70/30 7T system (Bruker, Ettlingen, Germany) with a birdcage head‐coil of 75 mm inner diameter for radio frequency (RF) transmission and a 20 mm diameter surface coil for reception were used. Prior to imaging, rats (n = 6) under anaesthesia (performed by initial inhalation of 4% isoflurane for 3 minutes and maintained with 2% isoflurane in a mixture of 20% oxygen and 80% air) were placed in the stereotaxic holder of MRI machine equipped with a heating system to maintain body temperature and a pressure detector to monitor respiration. PWIs were performed with pulsed arterial spin labelling (PASL) technique using a flow‐sensitive alternating inversion‐recovery echo planar imaging (FAIR‐EPI) sequence with matrix = 96 × 96, FOV = 25 × 25 mm^2^, inversion recovery time (TIR) = 30‐2300 ms, number of TIR values = 22, recovery time = 10 000 ms, TE/TR > = 10/18 000 ms. The T1 changes between slice selective inversion sequence and nonselective inversion sequence were used for CBF quantification at day 1, 3, 7, and 14. CBF ratio was measured by using PMOD software (version 3.0; PMOD Technologies).

### 2.9 Glucose metabolic evaluation by MicroPET/CT imaging

The glucose metabolism measured by ^18^F‐2‐deoxy‐glucose (FDG)‐PET/CT imaging was performed according to the protocol described in our previous report.^14^ Before imaging, SD rats in each group (n = 6) were fasted for 12 hours. After anaesthetized with isoflurane, rats were intravenously administered with 37 MBq (~1 mCi) of ^18^F‐FDG. One hour later, microPET/CT image was acquired for 30 minutes using a FLEX X‐PET and X‐O small animal imaging system (TriFoil) with the spatial resolution of 1.6 mm and the voxel size of 0.4 mm × 0.4 mm × 1.2 mm. CT images were acquired with 256 projections over 2 minutes for attenuation correction and anatomy landmarks. PET data were reconstructed using 3D ordered subset expectation maximization (OSEM) method. CT images were reconstructed using a cone‐beam reconstruction algorithm. PET and CT images were coregistered using commercial software (Visage Imaging) with 72‐μm isotropic CT spatial resolution and 2 mm for PET imaging. For quantitative analysis, regions of interests (ROIs) were drawn on the infarct lesion and at the mirror sites of the contralateral hemispheres. Per cent‐injected dose of ^18^F‐FDG per c.c. of brain tissue (%ID/cm3) was obtained from each ROI. The differential uptake ratio (DUR) of each ROI was calculated with the following formula: DUR = (%ID/c.c. of contralateral site ROI − %ID/c.c. of lesion site ROI)/%ID/c.c. of contralateral site ROI.

### 2.10 Evans blue dyeing and IVIS detection

Disruption of blood‐brain barrier (BBB) after photothrombotic stroke was evaluated by in vivo Evans blue (EB) fluorescent imaging.^14^ Briefly, 2 hours after EB dye (Sigma, 10 mg/mL in saline, 2.5 mL/kg rat weight) was injected via tail vein, animals in each group (n = 6) at day 3 and 7 were killled with an overdose of pentobarbital injection (RMB, Animal Health Ltd., UK). The brains were carefully removed from the skulls immediately after death. To detect the presence of EB, the intact brain was imaged using IVIS 50 (PerkinElmer, UK) with following steps and image acquisition settings: the brains were placed at the center of imaging field, and images were acquired for 2 seconds using Cy5.5 band pass filter channel (excitation/emission wavelength: 615~665 nm/695~770 nm). ROI selection and quantification were performed using living image software 3.2 (IVIS Imaging System, Perkin Elma, UK).

### 2.11 Statistical analysis

All results were expressed as means ± SD. One‐way ANOVA was performed to compare the protein expression (western blot and immunohistochemical staining), DURs, intensity of EB staining and rCBF among all treatment groups at different time‐points after stroke. A significant difference was considered when the p value was less than 0.05. All data were analysed using spss 19.0 (Chicago, IL, USA).

## 3 RESULTS

### 3.1 ADSC identification and luciferase gene introduction

**Figure 1:**
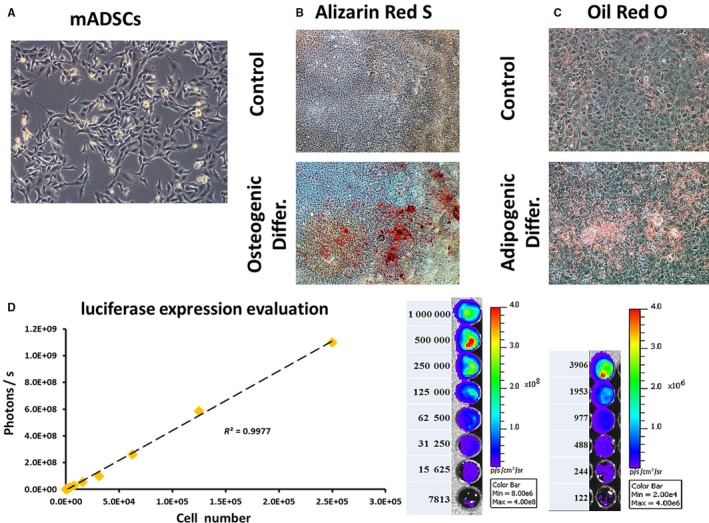
Characterization of ADSCs and luciferase expression evaluation. A, Cultured adipose‐derived adherent cells showed fibroblastic morphology. After induction of osteogenic and adipogenic differentiation, the cells were positively stained by Alizarin Red S and Oil Red O for the detection of bone matrix mineralization and lipid droplet, respectively (B and C). D, After lentiviral transduction of firefly luciferase gene, luciferase expression was evaluated by optical luminescence imaging and the signal was positively correlated with cell number

After isolation from mouse adipose tissue and a few passages of cell culture, the adherent stromal cells appeared as spindle fibroblastic morphology (Figure 1A). Mineralized bone matrix and lipid droplet were positively stained by Alizarin Red S and Oil Red‐O, respectively, after induction of osteogenic or adipogenic differentiation (Figure 1B,C). Control cells without induction of differentiation showed no reaction to both staining. These results indicate that the isolated adipose‐derived adherent cells possess the differentiation potential of MSCs, and were named as ADSCs in this study.

For tracking of ADSCs after injection into animals, Luc gene was introduced into ADSCs by lentiviral transduction followed by stable cell selection. Expression of Luc in ADSCs was imaged using an IVIS system. As shown in Figure 1D, expression of Luc in ADSCs was observed thru detecting the luminescence upon addition of luciferin. The intensity of luminescent signal in cells positively correlated with cell number (*R* ^2^ = 0.9977).

### 3.2 Photochemically induced thrombus in rats

**Figure 2:**
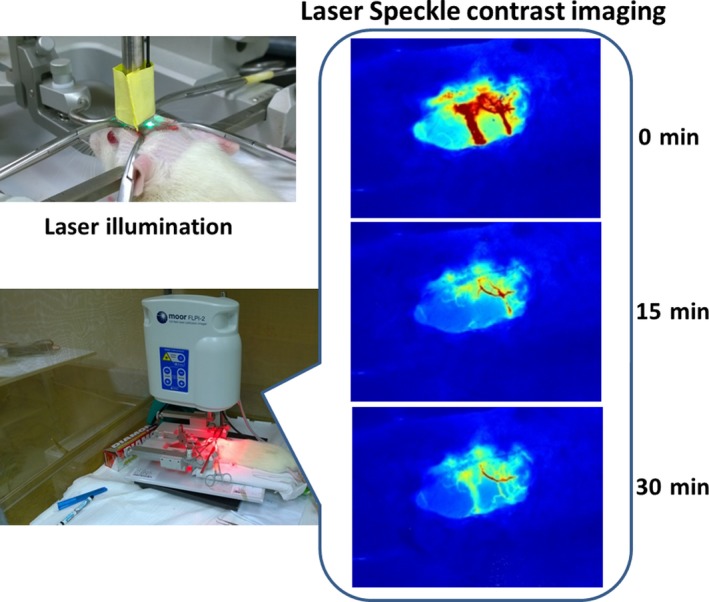
Photochemical induction of stroke. Photochemical induction of stroke was performed by intravenous injection of rose bengal followed by laser illumination at cranial window. Laser speckle contrast imaging revealed the blockage of blood flood and vasculature occlusion overtime

Ischaemic stroke was induced by photochemical method for subsequent treatment experiments. Vasoconstriction and blockade of blood flow were examined by laser speckle contrast imaging (LSCI). Before laser illumination, surface vessel network clearly observed at the cranial window, showing the uninterfered blood flow under normal physiological condition. After illumination for 15 minutes, obvious blockage and constriction were noted in the main vessel trunk as well as in the whole vascular network. After 30 minutes of illumination, overall blood flow was more severely blocked (Figure 2), indicating the successful induction of focal cerebral ischaemia in rats by PTS.

### 3.3 ADSC transplantation and tracking ex vivo

**Figure 3:**
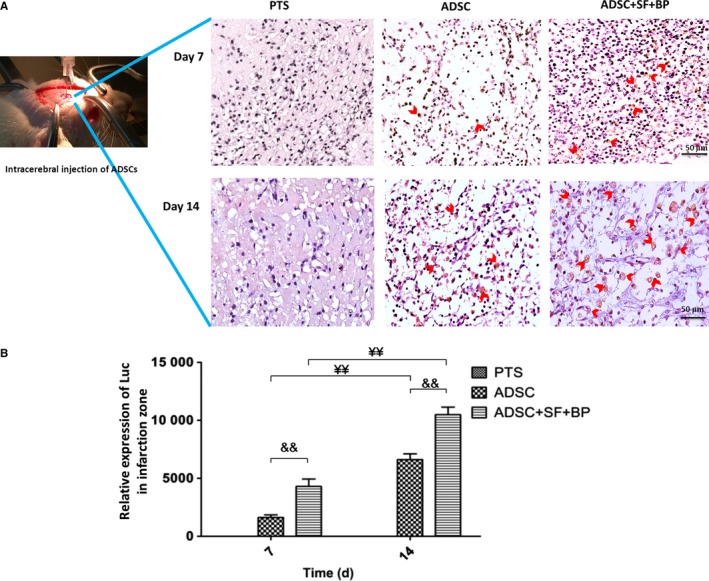
Fate of ADSCs after intracerebral transplantation in the infarct zone. A, Representative images of Luc^+^ ADSCs detected by immunohistochemical staining at day 7 and 14. B, Quantitative result suggested a favourable effect for ADSC survival and proliferation upon SF + BP treatment. Data are expressed as means ± SD. ^&&^ *P* < 0.01, compared with ADSC group; ^¥^ *P *< 0.05, ^¥¥^ *P *<0.01, compared with day 7. n = 6, scale bar: 50 μm

For ADSC treatment, cells were intracerebrally injected at the margin of ischaemic region after PTS. At day 7 and 14 after transplantation, Luc expression of ADSCs was detected in the brain of rats in group ADSC and ADSC + SF + BP (Figure 3A). Notably, the expression of Luc in group ADSC + SF + BP was higher than in group ADSC at both day 7 and 14, and also was significantly higher at day 14 than that at day 7 in the same group (Figure 3B, *P* < 0.01). This observation suggested that the microenvironment where the ADSCs were injected in the group treated with SF + BP might favour the survival of ADSCs, and even promote their proliferation.

### 3.4 Combined treatment of ADSCs, SF, and BF promote neovascularization in infarct lesion

**Figure 4:**
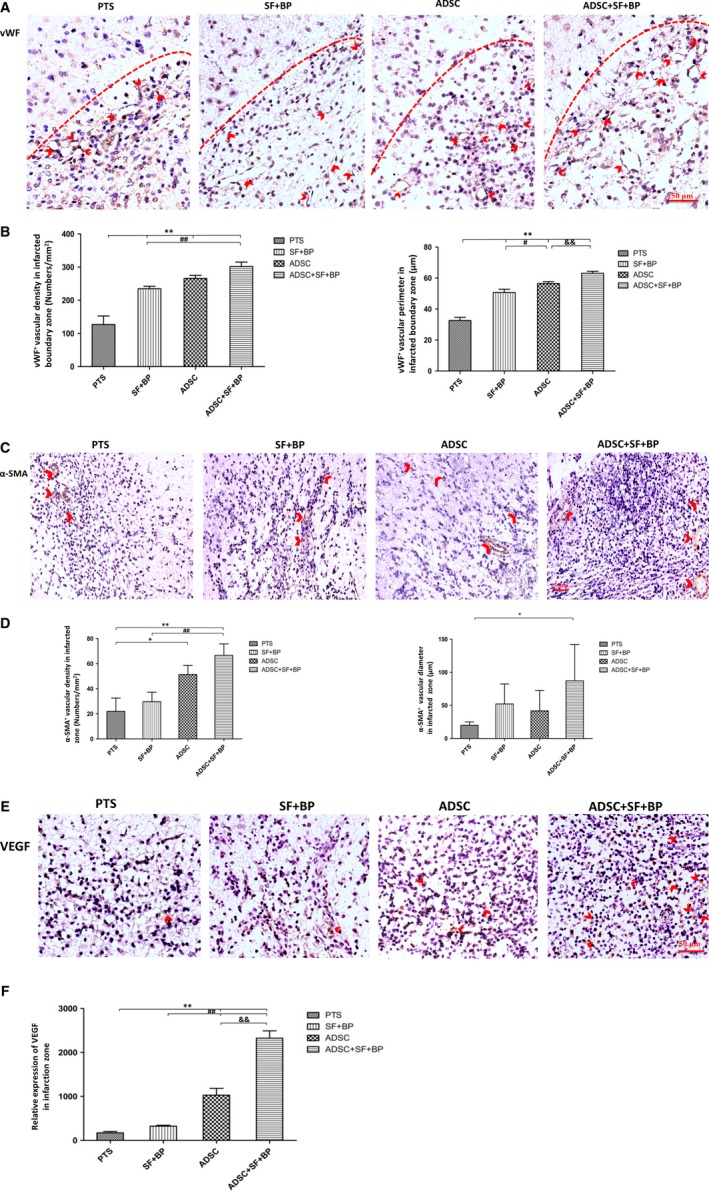
Evaluations of neovascularization and VEGF expression in the infarct lesion. Representative immunohistochemistry staining images expressed brown positive signals of vWF and α‐SMA, as well as VEGF were presented (A, C, and E). Quantitative results showed that ADSC + SF + BP treatment significantly enhanced the neovascularization and VEGF expression as compared with SF + BP or ADSC treatment alone (B, D, and F). Data were expressed as means ± SD. *P < 0.05, **P < 0.01, compared with PTS group; ^#^ *P* < 0.05, ^##^ *P *< 0.01, compared with SF + BP group; ^&^ *P *< 0.05, ^&&^ *P *< 0.01, compared with ADSC group. n = 6, scale bar: 50 μm

After treatment, the neovascularization was evaluated by detecting the expression of vWF and α‐SMA. As shown in Figure 4A,C, both vWF^+^ capillaries and α‐SMA^+^ vessels distributed in the infarct zone at day 7 poststroke. Quantitative analysis showed that the density of vWF^+^ capillary increased in group SF + BP and ADSC compared to that in group PTS (234.67 ± 7.64 and 265.32 ± 9.50/mm^2^ vs 126.47 ± 26.03/mm^2^, *P* < 0.01). The capillary density in group ADSC + SF + BP (301.62 ± 13.58/mm^2^) was significantly higher than that in group SF + BP (*P* < 0.01). The average capillary perimeter in group ADSC + SF + BP was 63.07 ± 1.28 μm, larger than that in ADSC group (56.49 ± 1.16 μm, *P* < 0.01) (Figure 4B). α‐SMA^+^ vascular density was 51.33 ± 7.23/mm^2^ in ADSC group and 66.67 ± 9.07/mm^2^ in ADSC+SF+BP group, which were 2.3‐fold and 3‐fold higher than that in PTS group (22.01 ± 10.54/mm^2^). The vascular diameter in group SF + BP was 52.08 ± 30.27 μm, which was larger than that in group ADSC (41.67 ± 30.81 μm) and group PTS (20.02 ± 5.03 μm). In group ADSC + SF + BP, the vascular diameter was 87.19 ± 54.76 μm, significantly higher than that in group PTS (*P* < 0.05) (Figure 4D). These results indicated that combined treatment of ADSC + SF + BP had better effect on vascularization including capillary/vascular density and diameter, as compared to treatment with ADSCs or SF + BP alone.

The expression of angiogenic factor VEGF was also evaluated, and the result showed that more cells with positive staining (brown) were presented in group ADSC and ADSC + SF + BP than in group SF + BP and PTS (Figure 4E). Quantitative result indicated that the expression of VEGF in ADSC group were 6‐fold and threefold higher than that in group PTS (*P* < 0.01) and SF + BP group (*P* < 0.01), respectively. The expression of VEGF in group ADSC + SF + BP were 2.3‐fold higher than that in group ADSC (*P* < 0.01, Figure 4F). These data showed that combined treatment of ADSCs and SF + BP effectively enhanced the expression of VEGF.

### 3.5 Combined treatment of ADSCs, SF, and BF modulate the activation of astrocytes and promote neurogenesis

**Figure 5:**
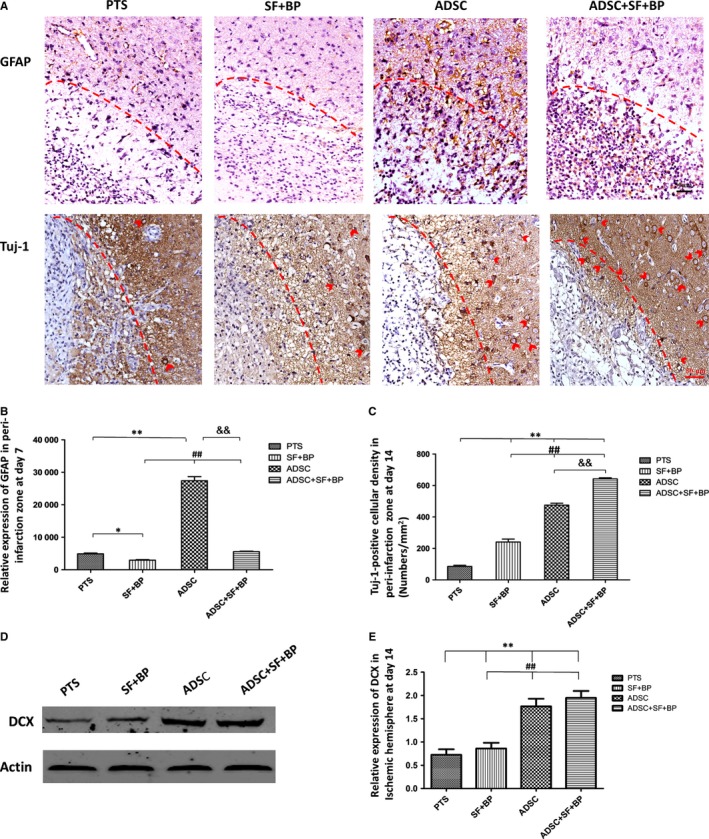
Modulation of astrocyte activation and neurogenesis after treatment. A, Representative images of GFAP and Tuj1 expression presented by immunohistochemical staining. B, The relative expression of GFAP and (C) the density of Tuj1+ cells in the infarct zone were calculated. D, Representative Western blot results for DCX in ischaemic hemisphere and quantitative analysis. Data are expressed as means ± SD. *P < 0.05, **P < 0.01, compared with PTS group; ^##^ *P *< 0.01, compared with SF + BP group; ^&&^ *P *< 0.01, compared with ADSC group. N = 6 (for immunohistochemical staining), N = 3 (for Western blot), scale bar: 50 μm

To know what the effect of ADSCs or SF + BP on astrocytes upon treatment of stroke, the expression of GFAP was detected by immunostaining. In group PTS, only few scattered GFAP^+^ cells were observed locating at the peri‐infarct zone, and even less number of cells were found in group SF + BP (Figure 5A). In contrast, obvious high number of GFAP^+^ cells was noted in group ADSC. Interestingly, in group ADSC + SF + BP, these GFAP^+^ cells mostly resided in the infarct region, instead of in the peri‐infarct region. Quantitative analysis showed that the number of GFAP^+^ cells in peri‐infarction zone in ADSC group was 5.6‐fold higher than in group PTS (*P* < 0.01), but was 1.7‐fold lower in group SF + BP group than in group PTS (*P* < 0.05, Figure 5B). These results suggested an inhibitory action on astrocyte activation by SF + BP treatment and an activation effect by ADSCs. Combined treatment by ADSC + SF + BP promoted the migration of active astrocytes into infarct region.

In order to assess whether combined treatment promoted the neurogenesis, immunohistochemical staining for Tuj1 and western blotting of DCX were performed. As shown in Figure 5A, Tuj1^+^ cells accumulated in the periphery of infarct region at day 14. The number of Tuj1^+^ cells in all treatment group ADSC + SF + BP, ADSC and SF + BP were significantly higher as compared with group PTS (642.33 ± 6.81, 475.43 ± 12.22 and 240.67 ± 18.58/mm^2^ vs 85.53 ± 7.51/mm^2^, *P* < 0.01, Figure 5C). Number of Tuj1^+^ cells in group ADSC + SF + BP was 1.4‐fold higher than group ADSC and 2.7‐fold higher than group SF + BP group (*P* < 0.01). Quantitative analysis of western blot showed that the expression of DCX in group ADSC + SF + BP at day 14 was the highest compared with those in other three groups (Figure 5D). The results suggested that there was a remarkable promotion of neurogenesis by combined treatment of ADSC + SF + BP.

### 3.6 The improvements of cerebral blood flow and glucose metabolism

**Figure 6:**
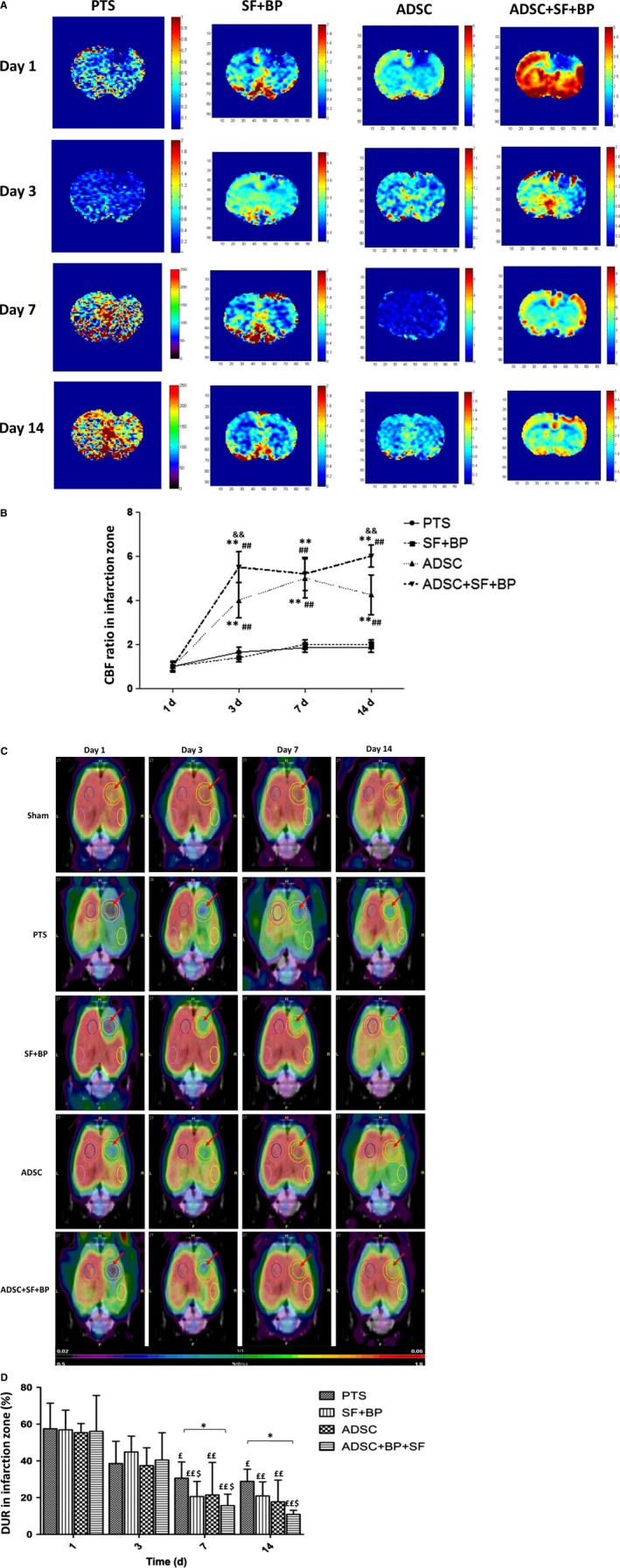
Evolutional change of cerebral blood flow and glucose metabolism in the infarct zone. (A) and (C) represent the images of PWI/MRI and ^18^F‐FDG/PET at day 1, 3, 7, and 14 after stroke with or without treatment. Quantitative analysis showed that ADSC + SF + BP treatment restored rCBF more rapidly B, and enhanced glucose metabolism more efficiently (C) as compared with other three groups. Data were expressed as means ± SD. *P < 0.05, **P < 0.01, compared with group PTS; ^##^ *P *< 0.01, compared with SF + BP group, ^&&^ *P *< 0.01, compared with ADSC group, ^£^ *P *< 0.05, ^££^ *P *< 0.01, compared with day 1, ^$^ *P *< 0.05, ^$$^ *P *< 0.01, compared with day 3. N = 6

To monitor the evolutional CBF change in photothrombotic stroke, PWI was performed on rats at day 1, 3, 7, and 14 after stroke induction (Figure 6A). Quantitative analysis indicated that the rCBF ratio at the infarct region in group ADSC and ADSC + SF + BP increased rapidly at day 3, with the value 2.4‐fold and 3.3‐fold higher than that in group PTS, respectively. At day 14, the ratios in both group increased and were 2.9‐fold and 3.9‐fold higher than that in group SF + BP (*P* < 0.01). Furthermore, at day 3 and day 14, rCBF ratio in group ADSC + SF + BP was higher than that in group ADSC (1.4‐fold at day 3, and 1.4 ‐fold at day 14, *P* < 0.01, Figure 6B). This result strongly suggested that combined treatment of ADSC and SF + BP significantly restored CBF in the infarct region after photothrombotic stroke.

^18^F–FDG PET imaging was performed for the investigation of metabolic change in each group at day 1, 3, 7, and 14 after stroke induction. As shown in Figure 6C, ^18^F‐FDG accumulation remarkably reduced in the infarct lesion in each group at day 1. The cortical metabolic defect gradually recovered over time, with much improvement observed in group ADSC and group ADSC + SF + BP at day 7 and 14. After calculation, DUR in group SF + BP, ADSC and ADSC + SF + BP declined from day 1 to day 7 (57.46% to 30.6% in group PTS, 56.92% to 20.65% in group SF + BP, 55.38% to 21.48% in group ADSC and 56.06% to 15.65% in group ADSC + SF + BP). DUR in each group at day 14 were 2.0‐fold, 2.7‐fold, 3.1‐fold and 5.1‐fold lower as compared with that at day 1 (*P* < 0.05, Figure 6D). Although the improvement of glucose metabolism in the infarct region was noted over time in each group, DUR in group ADSC + SF + BP was significantly improved than that in PTS group at day 7 (*P* = 0.034) and 14 (*P* = 0.038), showing that the combined treatment of ADSC + SF + BP effectively improved the glucose metabolism in infarct region.

### 3.7 Combined treatment of ADSCs, SF, and BF recovered BBB disruption

**Figure 7:**
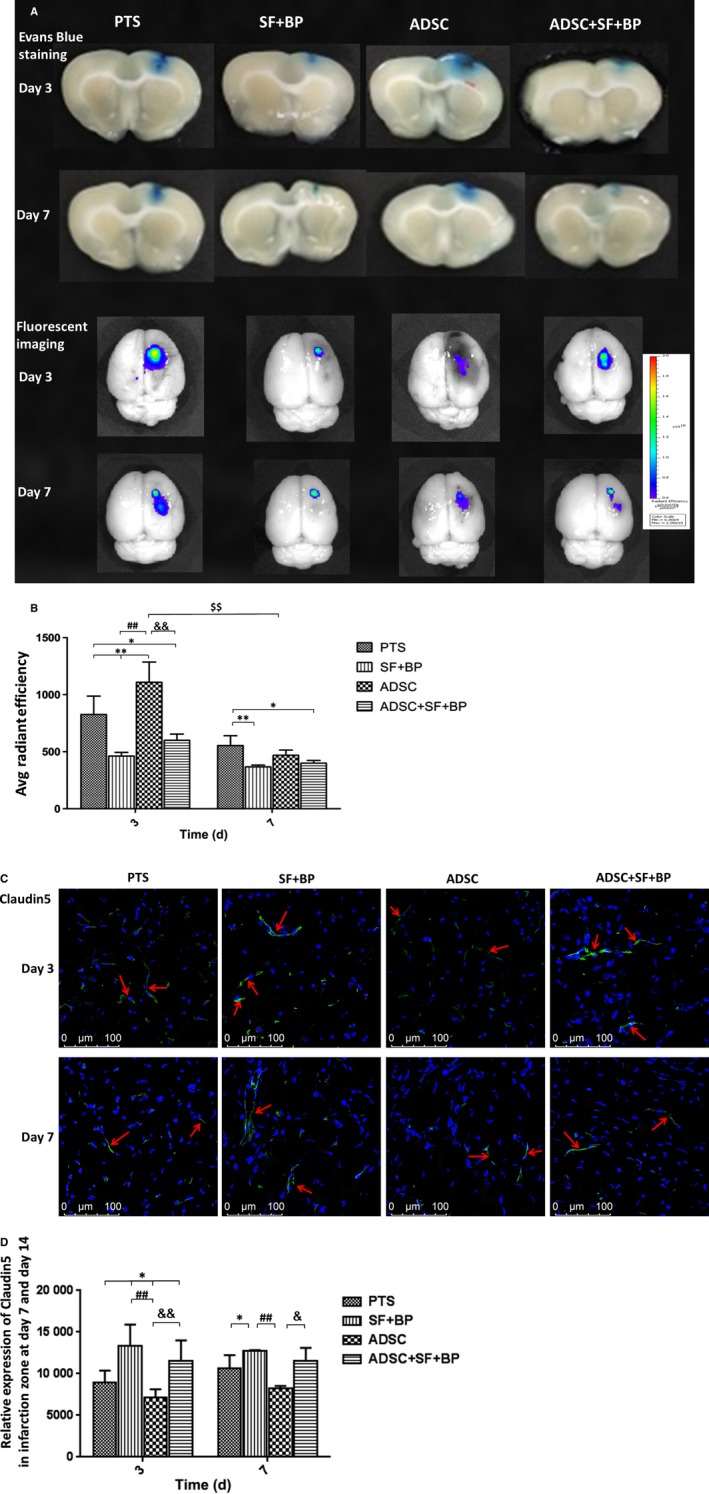
Evaluational change of blood‐brain‐barrier integrity poststroke. A, Representative images of EB staining and fluorescence imaging of rat brain after stroke in different treatment groups at day 3 and 7. B, Quantitative result of EB fluorescence showed that SF + BP treatment remarkably ameliorated BBB leakage while ADSC treatment had adverse effect at day 3. However, combined treatment of ADSC + SF + BP effectively ameliorated BBB leakage at day 3‐7. (C) and (D) Representative images of claudin‐5 by immunofluorescence staining were presented and quantitative analysis showed the expression of claudin‐5 in SF + BP group was the most significant. Data were expressed as means ± SD. *P < 0.05, **P < 0.01, compared with PTS group, ^##^ *P *< 0.01, compared with SF + BP group; ^&&^ *P *< 0.01, compared with ADSC group; ^$$^ *P* < 0.01, compared with day 3. N = 6. Scale bar: 100 μm

Our pervious study indicated that the leakage of BBB gradually increased at day 3 and began to restore at day 7 in rat PTS model.^14^ In this study, we sought to evaluate the restoration of BBB disruption after treatment. As shown in Figure 7A, Evans blue leakage was renovated, with greatest amelioration seen in group SF + BP. Treatment of ADSCs resulted in the highest BBB leakage even compared to group PTS at day 3. Quantitative analysis indicated that the fluorescence signal in group SF + BP was 1.8‐fold lower than that in group PTS at day 3 (*P* < 0.01), and signal in group ADSC was 1.3‐fold higher as compared with group PTS at day 3 (*P* < 0.01), suggesting that SF + BP treatment repaired the BBB leakage. At day 7, signal in group ADSC was still higher than in group SF + BP and ADSC + SF + BP, although there was no statistical difference (*P* > 0.05). However, at day 3 or 7, fluorescence signal in group ADSC + SF + BP was significantly lower than that in group ADSC group (Figure 7B). Additionally, as shown in Figure 7C, quantitative analysis of positive claudin‐5 (an important protein of tight junctions in BBB) signal (green fluorescent) in SF + BP group was significant higher than those in PTS group (*P* < 0.05) and ADSC group (*P* < 0.01) whatever at day 3 or day 7. The results suggested adverse effects on BBB leakage restoration from SF + BP and ADSCs, and also a net result of positive restoration by combined treatment of ADSC + SF + BP.

### 3.8 Regulations of AKT/mTOR, ERK1/2 and Notch1 signalling pathways

**Figure 8:**
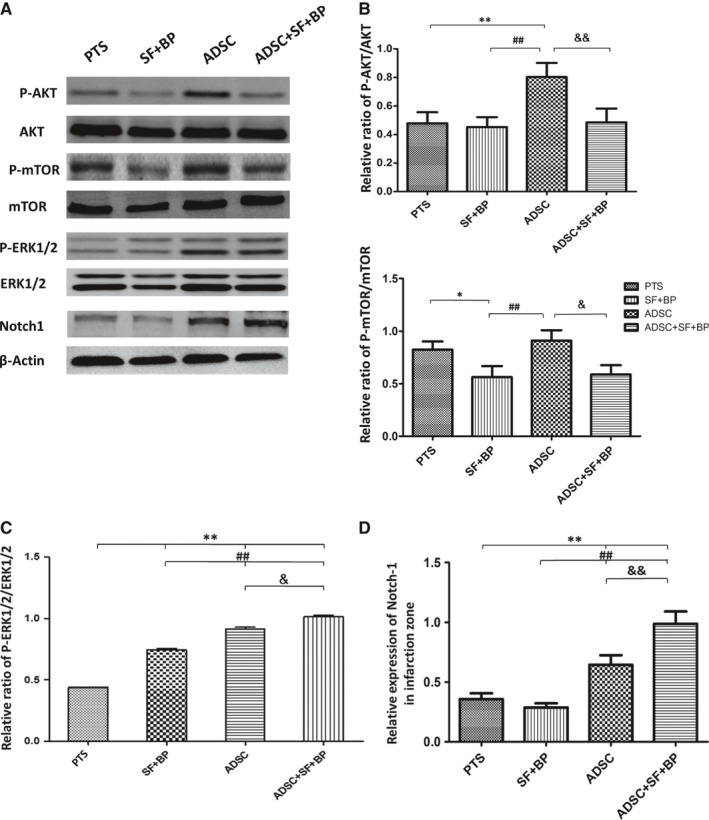
Regulations of AKT/mTOR, ERK1/2 and Notch1 signalling pathways in infarcted region. A, Result of western blot for p‐Akt, Akt, p‐mTOR, mTOR, p‐ERK1/2, ERK1/2, and Notch1. (B‐D) Quantitative analysis showed that combined ADSC + SF + BP treatment activated ERK1/2 and Notch1 cascades and suppressed AKT/mTOR signalling. Data are expressed as mean ± SD. *P < 0.05, **P < 0.01, compared with PTS group, ^##^ *P *< 0.01, compared with SF + BP group, ^&&^ *P* < 0.01, compared with ADSC group. N = 3

In order to explore the corresponding mechanism associated with NVU remodelling after combined treatment, AKT/mTOR, ERK1/2 and Notch1 pathway were analysed in the infarct region by western blot. As shown in Figure 8, phosphorylated AKT and mTOR in group ADSC was the most dominant compared with those in other three groups (*P* < 0.05), whereas phosphorylation of AKT and mTOR in group SF + BP decreased as compared with that in group PTS. Quantitative analysis indicated that phosphorylation level of AKT and mTOR in group ADSC + SF + BP fell between the level in group SF + BP and ADSC, suggesting that ADSC treatment activated AKT/mTOR pathway while SF + BP treatment had adverse effect. Phosphorylated ERK1/2 in group SF + BP and ADSC were higher than that in group PTS (*P* < 0.01), and combined treatment further enhanced ERK1/2 signalling (*P* < 0.01, vs SF + BP group; *P* < 0.05, vs ADSC group), showing an additive effect. Notch1 signalling in group ADSC and ADSC + SF + BP were notably activated as compared with that in group PTS (*P* < 0.01) and group SF + BP (*P* < 0.01). The result indicated that combined treatment was capable of activating ERK1/2 and Nothc1 cascade after ischaemic stroke.

## 4 DISCUSSION

In the present study, we have demonstrated the therapeutic effect of combined use of ADSC + SF + BP in the treatment of ischaemic stroke of rats induced by PTS and its related mechanism. Treatment of SF + BP was able to improve the survival of ADSCs and increase their proliferation. Combined treatment of ADSC + SF + BP promoted the neovascularization and neurogenesis, regulated the reactive astrocytes, enhanced rCBF and glucose metabolism in the infarct zone, and restored BBB leakage. AKT/mTOR, ERK1/2 and Notch1 cascades might be involved in the rehabilitation of NVU.

Among all the animal models of ischaemic stroke, PTS model stably produces a reproducible cortical photothrombosis with low mortality. In present study, in combination with optical infarct zone in MRI imaging and immunostaining, cortical artery occlusion model by photochemically embolism was successfully imitated.

Xenotransplantation of MSCs has been applied in several studies of different disease models and the results showed successful engraftment and functioning of transplanted xeno‐MSCs.^15^ Our results also showed a remarkable neovascularization and neurogenesis after xenotransplantation of mouse ADSCs in focal cerebral ischaemic rats, indicating the potential of cell therapy using xenogeneic‐derived stem cells.

In terms of the routes of cell transplantation in the treatment of ischaemic stroke, intravenous infusion is the most commonly used method because of its simple and safe procedure.^16^ It is hypothesized that the transplanted stem cells home and engraft in the injured site and subsequently exert the therapeutic effects, no matter by cell replacement or paracrine action. Several studies have proved that intravenously injected MSCs migrated to the brains of animal stoke, Alzheimer disease or glioma models.^17^ However, intravenously injected cells must pass through the pulmonary, systemic circulation and BBB, which greatly dilutes the cell amount arriving at the ischaemic site. Despite more invasive, intracerebral injection of MSCs ensures the cells to be preciously located at the lesioned brain with the maximum cell number.^18^ In a study, Kawabori et al. 19 clarified the favourable route of cell delivery for cerebral infarct in rats and concluded that intracerebral, but not intravenous, transplantation of BMSCs significantly enhanced functional recovery. Furthermore, the injected BMSCs widely distributed in the ischaemic brain and some of them expressed neural cell markers in the intracerebral group, but not in the intravenous group. Indeed, in our results, Luc^+^ cells remained detectable in group ADSC + SF + BP at day 14 after transplantation. These cells were found located at the peri‐infarct region and some had infiltrated in the infarct zone. Together with our results, SF + BP treatment further amplifies the efficacy of intracerebral transplantation of ADSCs which offers a lesioned site‐targeted, maximum cell number‐lodged way of cell therapy in ischaemic stroke.

It is now well accepted that upon acute ischaemic stroke, a rapid NVU injury is triggered. To restore NVU, reestablishment of vasculature in the infarct region is the major task upon treatment. A rebuilt vessel network will ensure the rCBF recovery, delivery of oxygen and nutrients, migration of stem/progenitor cells as well as removal of necrotic debris.^12^ In our result, we observed that combined treatment significantly increase neovascularization, as demonstrated by the increased number and diameter of vWF or α‐SMA‐positive vascular structure in the infarct region. Furthermore, the expression of VEGF in group ADSC + SF + BP was 2.3‐folds higher than that in group ADSC. These results indicated that combined treatment of ADSC + SF + BP had synergistic enhancement of neovascularization, as compare to the treatment of ADSC alone or SF + BP. With the increase in neovascularization after treatment, improvement of neurogenesis was also observed. By immunohistochemical staining and western blotting, we found that in group ADSC + SF + BP, the number of Tuj‐1+ cells in the infarct boundary and the expression of DCX in ipsilateral hemisphere were higher than those in ADSC group. In addition to participation in angiogenesis, the increasing VEGF by combination treatment also contributes to neurogenesis after stroke.^20^

Under pathological conditions (eg, stroke), it is reported that reactive astrocytes can modulate rCBF longer than neurons,^21^ and ADSCs can differentiate into astrocytes by the effect of autophagy and ADSC‐derived astrocytes have the function of clearing glutamate.^22^, ^23^ Additionally, Figure 5A showed that SF and BP facilitated astrocytes migration into infarcted zone. So we hypothesize that increasing astrocytes number in infarct region should participate in the enhancement of rCBF in group ADSC and ADSC + SF + BP. In the present study, rCBF in group SF + BP is notably lower than those in group ADSC and combination treatment, which might be related to inhibition of astrocytes activity. Of course, due to neovascularization, antiplatelet aggregation and anti‐oxidation activity of SF,^24^, ^25^ as well as antiplatelet effect of BP,^26^ rCBF in SF + BP group seems to slightly high in comparison with PTS group. Promotion of rCBF may subsequently lead to the transportation of oxygen and nutrients including glucose. To sum up, compared to sole treatment, combined treatment showed the best effects of neovascularization, neurogenesis and astrocytes activity, which led to the better restoration of rCBF and glucose metabolism in infarct region.

In terms of BBB integrity post stroke, we observed that SF + BP treatment distinctly reduced BBB leakage while ADSCs increased it. As a key integral protein that regulates BBB permeability,^27^ our result indicated that SF + BP group significantly increase claudin‐5 expression by immunofluorescence staining, which demonstrates that SF + BP is able to keep endothelial tight junction. In contrast, high rCBF led to increased cerebral perfusion pressure, as well as endothelial barrier of the newly formed vessels had greater instability, which aroused BBB permeability in ADSC group. Combined ADSC + SF + BP treatment did not exacerbate BBB leakage although it evidently improved rCBF, which should rely on endothelial tight junctions by SF + BP. It is reported that VEGF disrupts endothelial barrier and enhances BBB leakage.^28^ In our study, combination treatment did not exasperate BBB integrity, despite that VEGF expression was the highest among all the treatment groups. Recent report indicated that VEGF was able to bind by astrocyte‐derived Pentraxin 3, and subsequently decrease VEGF‐induced endothelial permeability in vitro.^29^ Some preclinical studies showed that BBB disruption poststroke did not totally attribute to tight junction disassembly, at least functional endothelial alterations and endothelial damage involved in the injury process.^30^, ^31^ Reeson et al^32^ demonstrated that VEGF‐associated BBB breakdown after stroke may be attributed to endothelial transcytosis rather than tight junctions in diabetic mice, but in nondiabetic mice, VEGF receptor 2 inhibitor increases BBB leakage. The studies reflect the multifaceted characteristics of VEGF on BBB integrity, so the precise mechanisms need to be further elucidated in the future.

Signalling of AKT/mTOR, ERK1/2 and Notch1 was reported to be at least partly involved in the BBB leakage, angiogenesis, neurogenesis, and reactive astrocytes upon ischaemia stroke.^13^, ^33^, ^34^ By suppression of Akt phosphorylation using electroacupuncture pretreatment in ischaemic cortices, BBB permeability together with brain oedema were able to be effectively reduced.^35^ Also, inhibition of mTOR by rapamycin increased the expession of tight junction protein zonula occludens‐1 in brain microvascular endothelial cells and attenuated EB extravasation in rat ischaemic hemisphere.^36^ In the present study, the inactivation of AKT/mTOR in the infarcted region by combination treatment was correlated with attenuation of BBB permeability. Notably, in our results, increased VEGF expression and angio‐neurogenesis after combined ADSC + SF + BP treatment may be associated with the activation of the ERK1/2 and Notch signalling. It was reported that activation of ERK1/2 cascade not only protected cortical neurons upon ischaemic injury, but also enhanced VEGF expression and angiogenic effect poststroke.^37^, ^38^ Activation of Notch signalling in human umbilical cord MSCs after cocultured with oxygen glucose deprivation‐induced neurons increased VEGF secretion and the ability of capillary‐like tube formation, as well as contributed to pericyte attachment and endothelial cells survival^39^, ^40^; simultaneously, activating signalling of Notch1 facilitated the subventricular zone neurogenesis after focal ischaemia.^41^ Furthermore, our study showed that SF + BP treatment significantly suppressed the reactive astrocytes, which correlated with its down‐regulated Notch1 signalling resulted in reduction in reactive astrocyte proliferation after ischaemic stroke.^42^

In summary, this is the first study demonstrating that combined treatment of SF + BP and ADSCs was able to effectively ameliorate the structure and function of the NVU in the infarct zone induced by photochemical stroke, and the therapeutic mechanism might associate with the regulations of AKT/mTOR, ERK1/2, and Notch1 pathways. These results may provide valuable information for developing future combined cellular and pharmacological therapeutic strategy for ischaemic stroke, especially targeting the NVU remodelling.

## CONFLICT OF INTEREST

The authors have declared that no conflict of interest exists.

## References

[1] Tuttolomondo A , Pecoraro R , Arnao V , et al. Developing drug strategies for the neuroprotective treatment of acute ischemic stroke. Expert Rev Neurother. 2015;15:1271‐1284. 26469760 10.1586/14737175.2015.1101345

[2] Lo EH , Dalkara T , Moskowitz MA . Mechanisms, challenges and opportunities in stroke. Nat Rev Neurosci. 2003;4:399‐415. 12728267 10.1038/nrn1106

[3] Maki T , Hayakawa K , Pham LD , et al. Biphasic mechanisms of neurovascular unit injury and protection in CNS diseases. CNS Neurol Disord Drug Targets. 2013;12:302‐315. 23469847 10.2174/1871527311312030004 PMC3845030

[4] Zhang ZG , Chopp M . Neurorestorative therapies for stroke: underlying mechanisms and translation to the clinic. Lancet Neurol. 2009;8:491‐500. 19375666 10.1016/S1474-4422(09)70061-4 PMC2727708

[5] Kowianski P , Lietzau G , Steliga A , et al. The astrocytic contribution to neurovascular coupling–still more questions than answers? Neurosci Res. 2013;75:171‐183. 23419863 10.1016/j.neures.2013.01.014

[6] Leu S , Lin YC , Yuen CM , et al. Adipose‐derived mesenchymal stem cells markedly attenuate brain infarct size and improve neurological function in rats. J Transl Med. 2010;8:63. 20584315 10.1186/1479-5876-8-63 PMC2913939

[7] Zhou F , Gao S , Wang L , et al. Human adipose‐derived stem cells partially rescue the stroke syndromes by promoting spatial learning and memory in mouse middle cerebral artery occlusion model. Stem Cell Res Ther. 2015;6:92. 25956259 10.1186/s13287-015-0078-1 PMC4453264

[8] Ikegame Y , Yamashita K , Hayashi S , et al. Comparison of mesenchymal stem cells from adipose tissue and bone marrow for ischemic stroke therapy. Cytotherapy. 2011;13:675‐685. 21231804 10.3109/14653249.2010.549122

[9] Li G , Yu F , Lei T , et al. Bone marrow mesenchymal stem cell therapy in ischemic stroke: mechanisms of action and treatment optimization strategies. Neural Regen Res. 2016;11:1015‐1024. 27482235 10.4103/1673-5374.184506 PMC4962565

[10] Wang Y , Deng Z , Lai X , et al. Differentiation of human bone marrow stromal cells into neural‐like cells induced by sodium ferulate in vitro. Cell Mol Immunol. 2005;2:225‐229. 16212891

[11] Liu SP , Harn HJ , Chien YJ , et al. *n*‐Butylidenephthalide (BP) maintains stem cell pluripotency by activating Jak2/Stat3 pathway and increases the efficiency of iPS cells generation. PLoS ONE. 2012;7:e44024. 22970157 10.1371/journal.pone.0044024 PMC3436873

[12] Zhang Q , Zhao YH . Therapeutic angiogenesis after ischemic stroke: Chinese medicines, bone marrow stromal cells (BMSCs) and their combinational treatment. Am J Chin Med. 2014;42:61‐77. 24467535 10.1142/S0192415X14500049

[13] Zhang Q , Zhao Y , Xu Y , et al. Sodium ferulate and n‐butylidenephthalate combined with bone marrow stromal cells (BMSCs) improve the therapeutic effects of angiogenesis and neurogenesis after rat focal cerebral ischemia. J Transl Med. 2016;14:223. 27465579 10.1186/s12967-016-0979-5 PMC4963939

[14] Liu NW , Ke CC , Zhao Y , et al. Evolutional characterization of photochemically induced stroke in rats: a multimodality imaging and molecular biological study. Transl Stroke Res. 2017;8:244‐256. 27910074 10.1007/s12975-016-0512-4 PMC5435782

[15] Li J , Ezzelarab MB , Cooper DK . Do mesenchymal stem cells function across species barriers? relevance for xenotransplantation Xenotransplantation. 2012;19:273‐285. 22978461 10.1111/xen.12000 PMC3445044

[16] Guzman R , Choi R , Gera A , et al. Intravascular cell replacement therapy for stroke. Neurosurg Focus. 2008;24:E15. 10.3171/FOC/2008/24/3-4/E14 18341391

[17] Liu L , Eckert MA , Riazifar H , et al. From blood to the brain: can systemically transplanted mesenchymal stem cells cross the blood‐brain barrier? Stem Cells Int. 2013;2013:435093. 23997771 10.1155/2013/435093 PMC3753739

[18] Jin K , Sun Y , Xie L , et al. Comparison of ischemia‐directed migration of neural precursor cells after intrastriatal, intraventricular, or intravenous transplantation in the rat. Neurobiol Dis. 2005;18:366‐374. 15686965 10.1016/j.nbd.2004.10.010

[19] Kawabori M , Kuroda S , Sugiyama T , et al. Intracerebral, but not intravenous, transplantation of bone marrow stromal cells enhances functional recovery in rat cerebral infarct: an optical imaging study. Neuropathology. 2012;32:217‐226. 22007875 10.1111/j.1440-1789.2011.01260.x

[20] Greenberg DA , Jin K . Vascular endothelial growth factors (VEGFs) and stroke. Cell Mol Life Sci. 2013;70:1753‐1761. 23475070 10.1007/s00018-013-1282-8 PMC3634892

[21] Girouard H , Iadecola C . Neurovascular coupling in the normal brain and in hypertension, stroke, and Alzheimer disease. J Appl Physiol. 1985;2006(100):328‐335. 10.1152/japplphysiol.00966.2005 16357086

[22] Sun Q , Ou Y , Wang S , et al. The effect of autophagy in the process of adipose‐derived stromal cells differentiation into astrocytes. J Mol Neurosci. 2014;53:608‐616. 24420732 10.1007/s12031-014-0227-5

[23] Cheng Z , Ou Y , Zhang L , et al. The glutamate clearance function of adipose stromal cells‐derived astrocytes. Neurosci Lett. 2018;677:94‐102. 29704575 10.1016/j.neulet.2018.04.048

[24] Hong Q , Ma ZC , Huang H , et al. Antithrombotic activities of ferulic acid via intracellular cyclic nucleotide signaling. Eur J Pharmacol. 2016;777:1‐8. 26948317 10.1016/j.ejphar.2016.01.005

[25] Qi D , Li Q , Chen C , et al. Ferulic acid modification enhances the anti‐oxidation activity of natural Hb in vitro. Artif Cells Nanomed Biotechnol. 2018;13:1‐9. 10.1080/21691401.2018.1448987 29533098

[26] Teng CM , Chen WY , Ko WC , et al. Antiplatelet effect of butylidenephthalide. Biochim Biophys Acta. 1987;924:375‐382. 3109495 10.1016/0304-4165(87)90151-6

[27] Lv J , Hu W , Yang Z , et al. Focusing on claudin‐5: a promising candidate in the regulation of BBB to treat ischemic stroke. Prog Neurobiol. 2018;161:79‐96. 29217457 10.1016/j.pneurobio.2017.12.001

[28] Argaw AT , Gurfein BT , Zhang Y , et al. VEGF‐mediated disruption of endothelial CLN‐5 promotes blood‐brain barrier breakdown. Proc Natl Acad Sci USA. 2009;106:1977‐1982. 19174516 10.1073/pnas.0808698106 PMC2644149

[29] Shindo A , Maki T , Mandeville ET , et al. Astrocyte‐derived Pentraxin 3 supports blood‐brain barrier integrity under acute phase of stroke. Stroke. 2016;47:1094‐1100. 26965847 10.1161/STROKEAHA.115.012133 PMC4811738

[30] Sandoval KE , Witt KA . Blood‐brain barrier tight junction permeability and ischemic stroke. Neurobiol Dis. 2008;32:200‐219. 18790057 10.1016/j.nbd.2008.08.005

[31] Krueger M , Hartig W , Reichenbach A , et al. Blood‐brain barrier breakdown after embolic stroke in rats occurs without ultrastructural evidence for disrupting tight junctions. PLoS ONE. 2013;8:e56419. 23468865 10.1371/journal.pone.0056419 PMC3582567

[32] Reeson P , Tennant KA , Gerrow K , et al. Delayed inhibition of VEGF signaling after stroke attenuates blood‐brain barrier breakdown and improves functional recovery in a comorbidity‐dependent manner. J Neurosci. 2015;35:5128‐5143. 25834040 10.1523/JNEUROSCI.2810-14.2015 PMC6705411

[33] Li W , Yang Y , Hu Z , et al. Neuroprotective effects of DAHP and Triptolide in focal cerebral ischemia via apoptosis inhibition and PI3K/Akt/mTOR pathway activation. Front Neuroanat. 2015;9:48. 25954164 10.3389/fnana.2015.00048 PMC4406066

[34] Zhang Q , Chen ZW , Zhao YH , et al. Bone marrow stromal cells combined with sodium ferulate and *n*‐Butylidenephthalide promote the effect of therapeutic angiogenesis via advancing astrocyte‐derived trophic factors after ischemic stroke. Cell Transplant. 2017;26:229‐242. 27772541 10.3727/096368916X693536 PMC5657770

[35] Zou R , Wu Z , Cui S . Electroacupuncture pretreatment attenuates bloodbrain barrier disruption following cerebral ischemia/reperfusion. Mol Med Rep. 2015;12:2027‐2034. 25936438 10.3892/mmr.2015.3672

[36] Li H , Gao A , Feng D , et al. Evaluation of the protective potential of brain microvascular endothelial cell autophagy on blood‐brain barrier integrity during experimental cerebral ischemia‐reperfusion injury. Transl Stroke Res. 2014;5:618‐626. 25070048 10.1007/s12975-014-0354-x

[37] Liu L , Zhang R , Liu K , et al. Tissue kallikrein protects cortical neurons against in vitro ischemia‐acidosis/reperfusion‐induced injury through the ERK1/2 pathway. Exp Neurol. 2009;219:453‐465. 19576887 10.1016/j.expneurol.2009.06.021

[38] Han L , Li J , Chen Y , et al. Human urinary Kallidinogenase promotes angiogenesis and cerebral perfusion in experimental stroke. PLoS ONE. 2015;10:e0134543. 26222055 10.1371/journal.pone.0134543 PMC4519127

[39] ElAli A , Theriault P , Rivest S . The role of pericytes in neurovascular unit remodeling in brain disorders. Int J Mol Sci. 2014;15:6453‐6474. 24743889 10.3390/ijms15046453 PMC4013640

[40] Zhu J , Liu Q , Jiang Y , et al. Enhanced angiogenesis promoted by human umbilical mesenchymal stem cell transplantation in stroked mouse is Notch1 signaling associated. Neuroscience. 2015;290:288‐299. 25637797 10.1016/j.neuroscience.2015.01.038

[41] Sun F , Mao X , Xie L , et al. Notch1 signaling modulates neuronal progenitor activity in the subventricular zone in response to aging and focal ischemia. Aging Cell. 2013;12:978‐987. 23834718 10.1111/acel.12134 PMC3838489

[42] Shimada IS , Borders A , Aronshtam A , et al. Proliferating reactive astrocytes are regulated by Notch‐1 in the peri‐infarct area after stroke. Stroke. 2011;42:3231‐3237. 21836083 10.1161/STROKEAHA.111.623280 PMC4469355

